# Impact of two different hesperidin forms loaded on nanoscale modified borate bioglass scaffolds on rat critical-sized calvarial defects

**DOI:** 10.1038/s41598-026-35881-z

**Published:** 2026-02-03

**Authors:** Nada A. Alqiran, A. M. Abdelghany, Samah Fouad, Mohammed E. Grawish, Yousry M. Elhawary, Eman Hany

**Affiliations:** 1 Department of Oral Biology, Faculty of Dentistry, Mansoura University, Mansoura, 35516 Egypt; 2 Spectroscopy Department, Physics Research Institute, National Research Center, 33 Elbehouth St., Dokki 12311, Cairo, Egypt; 3 Department of General and Basic Dental Sciences, Faculty of Dentistry, Horus University, International Coastal Road, New Damietta, Damietta, Egypt; 4 Medical Experimental Research Center (MERC), Faculty of Medicine, Mansoura University, Mansoura, 35516 Egypt; 5 Department of Oral Biology, Faculty of Oral and Dental Medicine, Delta University for Science and Technology, Dakahlia, Egypt

**Keywords:** Critical size, Calvarial defects, Modified borate bioglass scaffold, Hesperidin, Nanoscale, Osteopontin, Biotechnology, Materials science, Medical research, Nanoscience and technology

## Abstract

Bone defects remain a major clinical challenge because of their limited self-healing capacity. This study aimed primarily to evaluate whether nanoscale modified borate bioglass scaffolds (MBBGS) could enhance the delivery of hesperidin (HPN), an antioxidant flavonoid, to bone defects, and secondarily to compare the osteogenic effects of micro- and nano-sized HPN (mHPN and nHPN) on bone regeneration. Scaffolds were fabricated and fully characterized. Fifty-six healthy male albino rats were anesthetized, and bilateral critical-sized calvarial defects were created. The rats were divided into four groups: control (empty defects), MBBGS alone, MBBGS loaded with mHPN, and MBBGS loaded with nHPN. The animals were euthanized at the end of the 2nd and 6th weeks postoperatively. The skulls were harvested and processed for radiological, histological, and immunohistochemical analyses. Radiographic evaluation showed progressive defect closure over time, with the greatest reduction in defect size observed in the MBBGS + nHPN group compared to the other groups. Histologically, the MBBGS + nHPN group revealed the greatest osteogenic impact and significantly increased osteopontin (OPN) immunoreactivity, followed by MBBGS + mHPN and MBBGS groups, while the control group exhibited minimal bone healing and weak OPN reactivity. In conclusion, the newly fabricated MBBGS effectively promoted calvarial bone regeneration, which was further enhanced by loading with nHPN.

**Supplementary Information:**

The online version contains supplementary material available at 10.1038/s41598-026-35881-z.

## Introduction

The quality of life can be negatively impacted by bone abnormalities caused by aging, musculoskeletal conditions such as tumors, fractures, scoliosis, congenital malformations, osteoporosis, bone infection, and osteoarthritis, leading to large bone defects that do not heal on their own and necessitate medical intervention^1^. Finding effective bone regeneration techniques to promote the production of new bone is still a challenge on a global scale because bone defect healing is a dynamic and complex biological process^2^.

The most widely used technique for clinically treating bone defects is bone transplantation (including autologous and allogeneic grafts); however, it has some disadvantages, including a limited supply of donor tissue and the requirement for secondary surgery, which raises the risk of infection and surgical expenses^3^. To regenerate these defects, numerous researchers have developed a variety of biomaterials and procedures^4^. However, to determine their usefulness, these materials and procedures are often evaluated in vitro and in vivo before clinical application^5^.

Scaffolds are implants frequently utilized to introduce genes, medications, and cells into the body. Appropriate support for cell adhesion, proliferation, differentiated function, and migration is provided by their regular porous structure^6^. Scaffolds can be employed for a variety of tissue functions, such as bone production and periodontal regeneration. They are also used for the delivery of peptides, proteins, and growth factors, reflecting the growing interest in these applications within bone tissue engineering research^7^.

Biomaterial scaffolds remain a traditional method for bone regeneration because of their favorable mechanical qualities and acceptable degradation profile^8,9^. Biomaterials that resemble the structure of natural extracellular matrix can provide an environment that resembles bone, control cellular behaviors such as adhesion, proliferation, migration, and differentiation, and increase bone regeneration by utilizing the synergistic action of cytokines^10^.

A critical-sized defect (CSD) in rat calvaria is commonly used as an in vivo model for bone regeneration. The use of biomaterials is trending away from conventional bio-inert materials toward a new generation of bioactive materials, like bioactive glass scaffolds, which are used to repair CSD, especially in structural bone^11^. The capacity of bioactive glass to promote osteogenesis is attributable to its ability to form a hydroxyapatite (HA) layer, which bonds to bone tissue and releases ions such as calcium and phosphorus that stimulate osteoblasts. Additionally, it forms a strong connection with soft tissues^12,13^. However, glass-based scaffolds also have limitations, such as brittleness, decreased mechanical strength at high porosity, and difficulties in managing degradation behavior^14^.

Over the past few years, many studies have shown that certain borate glass compositions are bioactive when compared to silicate 45S5 glass. When borate glasses are immersed in an aqueous phosphate solution in vitro, they convert to HA at a controlled rate^15,16^. Bioactive borate glasses are thought to be converted to HA in bodily fluids through a dissolution–precipitation mechanism like that of silicate bioactive glasses, but without the silica-rich layer first forming^17^.

Calcium phosphates, natural and synthetic polymers, and HA form a scaffold that satisfies the requirements for biocompatibility and bioactivity^18^. To produce porous, permeable scaffolds, different methods such as sol-gel processing, solidification drying, and particle leaching were employed^19^. Compared to natural materials, synthetic materials have more flexible qualities, unlimited shapes, and sturdy structures^20^. The sol-gel-derived borate bioglass, a synthetic inorganic material, demonstrated remarkable healing properties when applied to wound lesions^21^.

A class of polyphenolic substances called flavonoids is present in plant cells. Among these interesting compounds, hesperidin (HPN) is a bioflavonoid found in citrus fruits^22^. It was found that HPN can alleviate colitis in model rats by lowering the levels of inflammatory factors, antioxidation, and mucosal cell apoptosis^23^. A previous study showed notable wound-healing capabilities of HPN; however, its low solubility and systemic absorption hinder its bioavailability when given topically. Therefore, appropriate dosage and formulations are required to increase its therapeutic efficacy^24^.

Nanoscale modifications of bioglass scaffolds have been shown to enhance surface reactivity and drug-loading efficiency, potentially optimizing the localized delivery of HPN. However, the interplay between scaffold nanostructure and HPN molecular form in bone defect healing has not yet been systematically examined^25,26^. HPN can be incorporated into scaffolds as micro- and nano-sized forms (mHPN and nHPN, respectively), each possessing distinct physicochemical properties such as surface area, dissolution rate, and dispersibility, while the chemical structure of HPN remains unchanged^27^. Despite this, the comparative effects of different HPN forms when combined with borate bioglass scaffolds still need to be explored.

This study examined the impact of mHPN and nHPN forms loaded on nanoscale modified borate bioglass scaffolds (MBBGS) for treating CSD in rat calvaria. We hypothesized that the form of HPN would significantly affect its release kinetics, cellular responses, and in vivo bone regeneration when combined with MBBGS. By analyzing radiographic, histological, and immunohistochemical metrics of bone formation, this work aimed to determine the best form of HPN for bone tissue engineering, advancing the development of flavonoid-enhanced bioactive scaffolds.

## Materials and methods

### Materials

Scaffolds were fabricated from pure chemical-grade materials. Boron and phosphorus precursors were boric acid (H_3_BO_3_) and phosphoric acid (H_3_PO_4_), respectively. Calcium and sodium were obtained from calcium nitrate tetrahydrate (Ca (NO_3_)_2_.4H_₂_O) and sodium nitrate (NaNO_3_), respectively. To catalyze hydrolysis, hydrochloric acid (HCl) was added to a solvent mixture of ethanol (C_2_H_5_OH) and deionized water. HPN drug and simulated body fluid (SBF) were used for drug loading and in vitro bioactivity evaluation.

### Scaffolds preparation

#### Preparation of MBBGS

The preparation of MBBGS (45B_2_O_3_-24.5CaO-24.5Na_2_O-6P_2_O_5_ mol%) was synthesized using the sol-gel method^28^. The process involved dissolving precursors in a water-ethanol mixture. Hydrolysis and condensation formed a sol, which transitioned into a gel. The gel was aged and carefully dried to prevent cracking, then calcined at about 650 °C to remove residual organic compounds and nitrates, and to initiate the formation of the glass structure. Finally, sintering at higher temperatures (around 1100 °C) was performed to achieve the desired density and crystallinity of MBBGS suitable for drug loading. The scaffold height was 1 mm, designed to fit precisely within the CSD and ensure complete filling and uniform contact with the surrounding bone.

#### Preparation of mHPN loaded on MBBGS

A wet impregnation method was used to load the purchased mHPN onto the sintered MBBGS^29^. The sintered porous scaffold was fully submerged in a 2 mg/mL HPN solution (ethanol: water 70/30 v/v) and maintained under gentle stirring at room temperature for 24 h in light-protected conditions, to allow drug diffusion and adsorption throughout its porous network^30,31^. Afterwards, the scaffold was removed, gently rinsed with ethanol to eliminate loosely bound surface drug, and dried under vacuum at 45 °C for 12 h to yield the mHPN-loaded scaffold.

#### Preparation of nHPN loaded on MBBGS

Nanoprecipitation was used for nHPN preparation and subsequently loaded onto the sintered MBBGS scaffold via wet impregnation and dropwise technique^32^. HPN was dissolved in ethanol and precipitated into water to form nanoparticles, which were ultrasonicated using probe sonication for 10 min to ensure uniform dispersion. The porous MBBGS scaffold was then immersed in the freshly prepared nHPN suspension and kept under gentle stirring for 24 h to enable nanoparticle infiltration into its interconnected pores. Following incubation, the scaffold was carefully removed, rinsed with deionized water to remove unbound nanoparticles, and vacuum dried at 40^°^C to obtain the nHPN-loaded scaffold.

### Scaffolds’ characterization

The surface morphology of the prepared scaffolds was examined by scanning electron microscopy (SEM) analysis. They were coated with a thin layer of gold, and the microscope was operated at an accelerating voltage of 30 KV, a working distance of 11 mm, and a magnification of ×20,000 at the Electron Microscopy Unit, Mansoura University, Egypt.

The crystallographic structure of the materials was determined by X-ray diffraction (XRD) analysis that was taken with the help of a diffractometer, occupied with Cu ά radiation at ƛ = 1.540° A, with a tube operating voltage of around 30 kV, Bragg’s angle (2θ) ranging from 5° to 80° and the X-ray runs were carried out at scanning speed of 2°/min.

To evaluate the chemical composition of the prepared samples, Fourier transform infrared (FTIR) spectroscopy analysis was recorded for various scaffolds using single-beam spectrometers in the spectral range (4000 − 500) cm^− 1^ with a resolution of 2 cm^− 1^.

### Evaluation of the bioactivity of scaffolds in vitro

The bioactivity of the glass was assessed in vitro from the conversion of the fabricated scaffolds after immersing them in Kokubo’s SBF (pH 7.4, 37 °C), formulated to simulate human plasma ion concentrations^33^. Before immersion, scaffolds were weighed and then submerged in SBF at a fixed ratio (e.g., 50 mg scaffold/50 mL SBF) and incubated under gentle agitation. The SBF solution was refreshed every two days to maintain ionic stability and prevent saturation. At predetermined time intervals up to 30 days, samples were retrieved, rinsed with distilled water, dried at 60 °C for 24 h, and reweighed to calculate percentage weight loss^34^. The degradation rate was further characterized after 30 days using SEM to observe surface morphology. An energy dispersive X-ray (EDX) spectrometer, integrated with AZtec software (Oxford Instruments; https://www.oxinst.com), was used to determine elemental composition.

### In vivo studies

#### Sample size and animal subjects

Sample size was calculated using the G*Power software (Version 3.1.9.4; https://www.gpower.hhu.de) program for a fixed-effects ANOVA with two factors. The input parameters were 0.40 for the effect (f), 0.05 for α error probability, 0.95 for the power analysis (1-β error probability), and 8 for the number of groups (4 groups with 2 examination periods), resulting in approximately 112 defects. According to that, a total number of 56 male pathogen-free Albino rats, weighing 300–350 g, were included in the present study, each receiving bilateral CSDs in the calvaria.

#### Study design

**Fig. 1:**
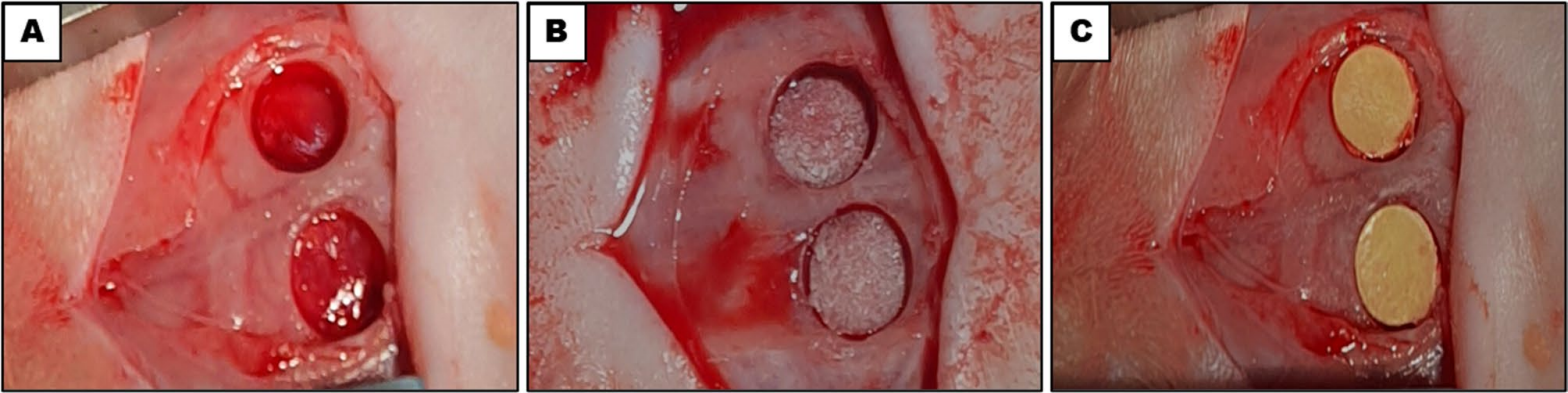
Bilateral rat critical-sized calvarial defects were created and assigned to four experimental groups: (**A**) control group, (**B**) MBBGS group, and (**C**) MBBGS loaded with HPN (mHPN or nHPN) groups.

Rats were divided into four groups in this study: (1) an untreated control group, (2) an MBBGS group, (3) an MBBGS loaded with mHPN (MBBGS + mHPN) group, and (4) an MBBGS loaded with nHPN (MBBGS + nHPN) group. Eight experimental groups were created by further dividing each group into two healing time periods (2 and 6 weeks). Each group had 14 calvarial defects obtained from 7 rats (2 defects per rat). All rats underwent the same surgical procedures. The control group received no implant, allowing for natural healing (Fig. 1A). The second group received MBBGS alone (Fig. 1B), while the third and fourth groups received MBBGS loaded with mHPN and nHPN, respectively, which had nearly identical clinical appearances (Fig. 1C).

#### Surgical procedures, postoperative care, and euthanasia

All surgical procedures were done under aseptic conditions in an operating room in Mansoura Experimental Research Centre (MERC), following a previous protocol^35^. Rats were obtained from the animal house of MERC. The animals received a standard pelleted diet and water ad libitum and were maintained on a 12-h light/dark cycle. They were acclimated for two weeks prior to the experiment. All experimental procedures were performed according to a protocol approved by the Ethical Committee of the Faculty of Dentistry, Mansoura University, Egypt (No. A03080622), and were in accordance with relevant institutional and national guidelines and regulations. All procedures followed the reporting ARRIVE guidelines for animal experiments (https://www.arriveguidelines.org/).

First, the rats were anesthetized, then shaved, and the surgical area was disinfected with 10% povidone-iodine. After that, a 1.5-cm sagittal incision was made in the scalp, and the calvarium was exposed by blunt dissection. Bilateral circular full-thickness CSDs were then created, ensuring that the dura mater wasn’t disturbed. Scaffolds were precut with a trephine bur (5 mm diameter) to match the defect size and were sterilized with low-energy UV irradiation (254 nm for 2 h) before placement, following a previous protocol^36^. Finally, the wound was sutured in layers as described in a previous study^37^.

Postoperative care was provided as previously described^38,39^. At the designated time points, rats were euthanized by an overdose of sodium thiopental, and the skulls were harvested for radiological and histological examinations.

### Cone-beam computed tomography (CBCT)

According to the examination periods, high-resolution CBCT scans of the rats’ skulls were performed using the imaging system, operated at a high-resolution voxel size of 0.1 mm, with a tube voltage of 120 kV, a current of 5 mA, and an integration time of 26.9 s. The mandibular condyles were used as bilateral reference markers for standardizing coronal orientation for anatomical uniformity across all samples. A standardized rectangular region of interest (ROI) involving 12 × 36 mm was positioned bilaterally within the coronal sections, covering the entire defect area to assess bone healing. The acquired datasets were analyzed using OnDemand3D software (Version 1.0.10.7510; https://www.ondemand3d.com) for standardized bone regeneration assessment and anatomical landmark identification. CBCT slice thickness was 1 mm, matching the depth of CSD. The grayscale values were interpreted semi-quantitatively, and no phantom was used for grayscale calibration.

### Histological examination

Following sample collection, all specimens were fixed in 10% neutral buffered formalin for 24 h and subsequently decalcified using 10% EDTA with frequent agitation. Completion of decalcification was confirmed by needle probing. The tissues were then processed using the paraffin embedding technique^40^. Hematoxylin and Eosin (H&E) staining was performed to assess general tissue morphology. Masson’s Trichrome staining (MTC) was used to evaluate collagen fiber distribution and bone matrix organization, where collagen and osteoid tissue appeared blue and mineralized bone appeared red. Immunohistochemical staining for osteopontin (OPN), an early osteogenic marker, was performed to evaluate osteogenic activity using the HRP/DAB Detection System kit, resulting in a brown precipitate at antigen sites.

### Slides imaging and digitizing

Slides were thoroughly examined and photographed using a digital camera mounted on a light microscope, equipped with a 0.5x photo adapter, and imaged at ×40 and ×100 magnifications in the Oral Biology Department, Faculty of Dentistry, Mansoura University. The images were analyzed with a specific built-in routine for measuring defined areas.

For histomorphometric standardization, five non-overlapping fields (1 × 1 mm^2^) per section were analyzed at ×100 magnification: one at the center of the defect and four at its boundaries. Histomorphometric measurements of collagen content (percentage of collagen-stained area, MTC) and OPN expression (percentage of positively stained area) were obtained for each field, and the mean value of the five fields was used to represent each section using standardized thresholding. All analyses were performed by a blinded examiner using ImageJ Fiji software (Version 2; https://www.fiji.sc).

### Supplementary information

Detailed information on chemicals used for in vitro experiments, equipment and manufacturers, medications, stains, antibodies, and immunohistochemical staining protocol was provided in supplementary data (Tables S1-S4).

### Statistical analysis

All data were collected and analyzed using GraphPad Prism software (Version 8.1; https://www.graphpad.com). Data were presented as mean ± standard deviation (SD). Normality of distributions was assessed using the Shapiro–Wilk test. For intergroup comparisons at each time point, two-way analysis of variance (ANOVA) was conducted to evaluate the effects of group and time on the measured parameters (CBCT, collagen content (MTC), and OPN expression), followed by pairwise independent t-tests for group comparisons. Intragroup comparisons across time points (2 weeks vs. 6 weeks) were evaluated using paired t-tests. Results were graphically presented as charts.

## Results

### Scaffolds’ characterization

**Fig. 2:**
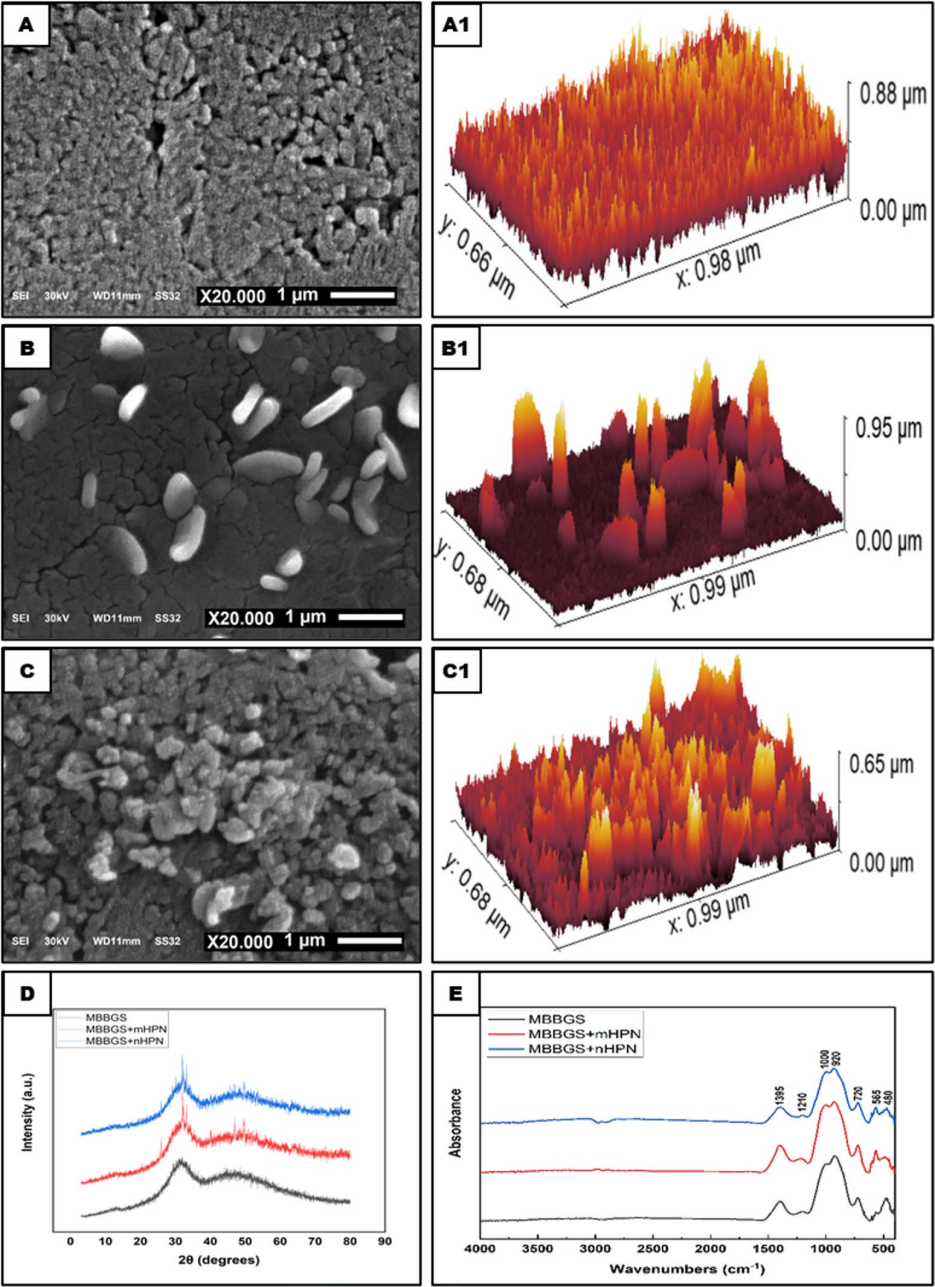
Characterization of different scaffolds. SEM micrographs and their corresponding 3D images of MBBGS (**A**,**A1**), MBBGS + mHPN (**B**,**B1)**, and MBBGS + nHPN (**C**,**C1**). XRD patterns of the three scaffolds (**D**). FTIR spectra of the scaffolds (**E**).

The morphology of the scaffold surface was visualized by SEM, as the parent MBBGS (Figs. 2A, A1) revealed a primarily smooth, spongy topography with minimal surface texture, producing a somewhat varying relief pattern and indicating well-processed glass. Upon incorporation of mHPN into the glass surface (Figs. 2B, B1), a small quantity of rod-shaped particles appeared on the predominantly smooth glass surface. Their localized distribution might indicate minimal surface interaction with the environment. The sol-gel process, including sintering and drying, could result in the appearance of a cracked underlying base. Notably, when nHPN was added to the MBBGS (Figs. 2C, C1), a uniform distribution of small aggregates across the underlying glass surface appeared, indicating a consistent modification process that contributed to the enhancement of surface energy and modified optical properties compared to the pristine base. Since this unique surface morphology constituted a controlled surface alternation at the nanoscale level, it was of interest for applications in materials science.

The X-ray diffraction pattern (Fig. 2D) revealed distinctive structural characteristics and phase transformations in the borate bioglass system with different forms of HPN incorporation. The base borate bioglass exhibited a characteristic amorphous structure, evidenced by two broad diffraction halos centered at Bragg angles of approximately 30° and 48°. The absence of long-range crystalline periodicity and the presence of short-range order in the glass were commonly shown by these broad halos. When mHPN was added, sharp crystalline peaks emerged in the diffraction pattern, while maintaining the glass’s underlying amorphous nature. Alternatively, when nHPN was incorporated, relatively low-intensity crystalline peaks emerged superimposed on the underlying MBBGS, preserving the predominantly glassy matrix, and providing unique qualities useful for biomedical applications.

The FTIR spectra of the three different scaffolds (Fig. 2E) revealed that the key structural features of MBBGS were influenced by the incorporation of mHPN and nHPN. The broad peak at 1395 cm⁻¹ corresponded to B-O stretching vibrations in trigonal BO₃ units, indicating the presence of borate networks. The bands at 1210 cm⁻¹ and 1000 cm⁻¹ were attributed to B-O stretching in tetrahedral BO₄ units and B-O-B/P-O stretching vibrations, respectively, suggesting phosphate and borate integration. The peak at 920 cm⁻¹ was associated with B-O-B bending or P-O-P vibrations, while the 720 cm⁻¹ band signified symmetric stretching of P-O-P bridges in phosphate chains. The lower-frequency peaks at 565 cm⁻¹ and 480 cm⁻¹ arose from bending modes of O-P-O and metal-oxygen (Ca-O/Na-O) vibrations, respectively.

In the MBBGS + mHPN and MBBGS + nHPN samples, the intensified peaks at 1000 cm⁻¹ and 920 cm⁻¹ suggested enhanced cross-linking between borate and phosphate networks, likely due to hydroxyl groups of HPN forming hydrogen bonds with the glass matrix. The shoulder in the ascending slope of these peaks indicated structural distortion, possibly arising from the higher surface area of nHPN, which induced greater network disruption. The increased intensity in the 400–650 cm⁻¹ region reflected stronger metal-oxygen interactions, implying that nHPN promoted more homogeneous ion distribution. The smaller size of nHPN likely facilitated deeper penetration into the glass network, creating more defects and increasing vibrational modes, whereas mHPN might cause localized stress, broadening peaks without significant structural refinement. Overall, nHPN induced more pronounced modifications, enhancing bioactivity via improved network connectivity and ion release.

### Degradation and conversion of the glass scaffolds in vitro

**Fig. 3:**
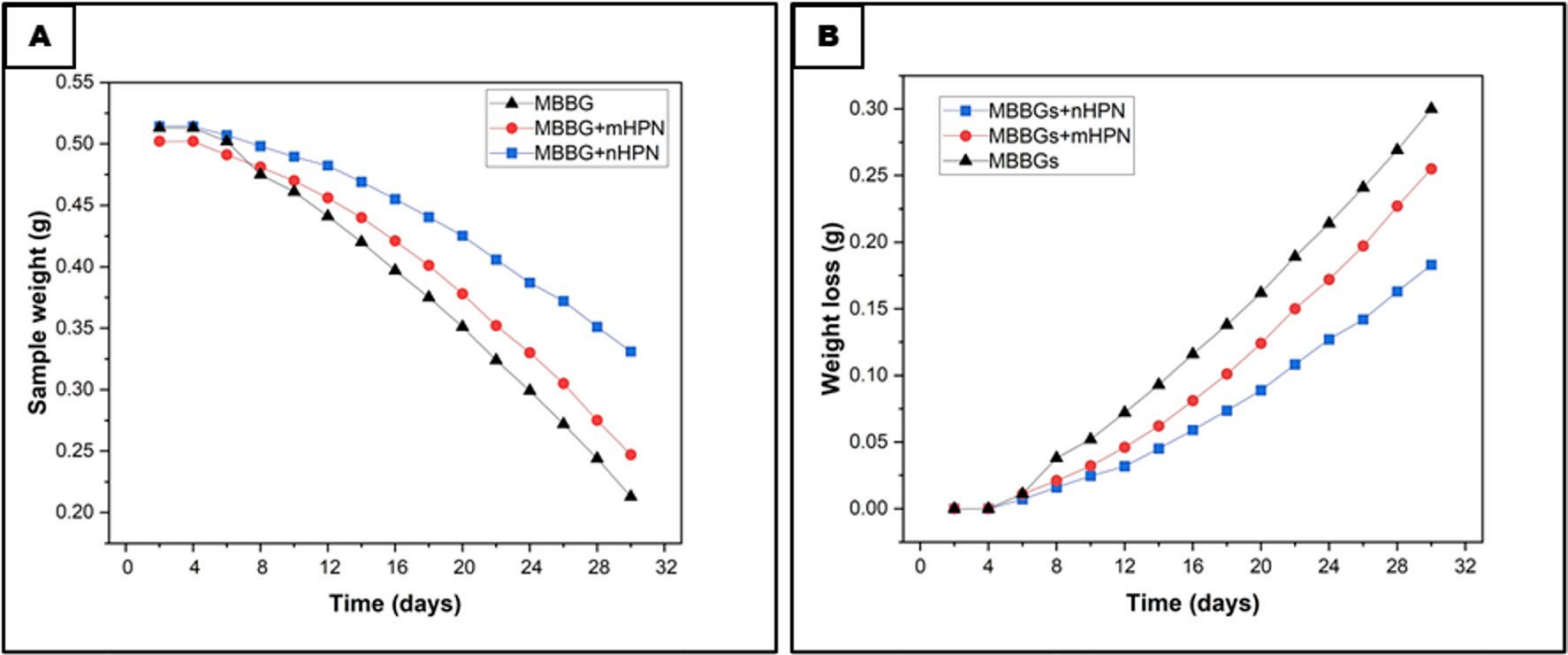
Degradation of different scaffolds after immersion in SBF for different time intervals up to 30 days, which showed an inverted decreasing weight exponential trend (**A**), and an inverted exponential decay pattern (**B**).

The degradation behavior of the three different scaffolds revealed critical insights into their structural stability and dissolution kinetics when they were immersed in SBF over a period of 30 days. The weight loss data followed an inverted decreasing exponential trend (Fig. 3A), indicating that degradation occurred rapidly in the initial stages before gradually slowing down. The MBBGS exhibited the highest degradation rate, with its weight decreasing from 0.52 to approximately 0.20, suggesting a high solubility of the borate network in aqueous media. This behavior was typical for borate-based glasses, where the rapid hydrolysis of B-O-B bonds led to swift ion release and matrix breakdown.

MBBGS + mHPN showed a slightly reduced degradation rate, with its weight declining to approximately 0.22, implying that the incorporation of mHPN provided marginal stabilization, likely due to weak interactions between the organic component and the inorganic glass network. In contrast, MBBGS + nHPN demonstrated the slowest degradation, retaining approximately 0.35 of its initial weight after 30 days. This significant retardation in dissolution could be attributed to the nanoscale dispersion of HPN, which enhanced interfacial bonding with the glass matrix, thereby reinforcing structural integrity. The higher surface area of nHPN allowed for more uniform integration, creating a protective barrier that slowed ion leaching and glass network breakdown.

The inverted exponential decay pattern (Fig. 3B) suggested that degradation was initially controlled by surface reaction (rapid dissolution of weakly bonded species), followed by diffusion-limited kinetics as a silica-rich or HPN-stabilized layer forms, hindering further dissolution. The nHPN composite’s superior retention of mass underscored its potential for controlled degradation in biomedical applications, where sustained ion release and mechanical stability were crucial for bone regeneration. These findings highlighted that nanoscale modification significantly enhanced bioglass durability compared to microscale additives, making it a more promising candidate for long-term implantable scaffolds.

**Fig. 4:**
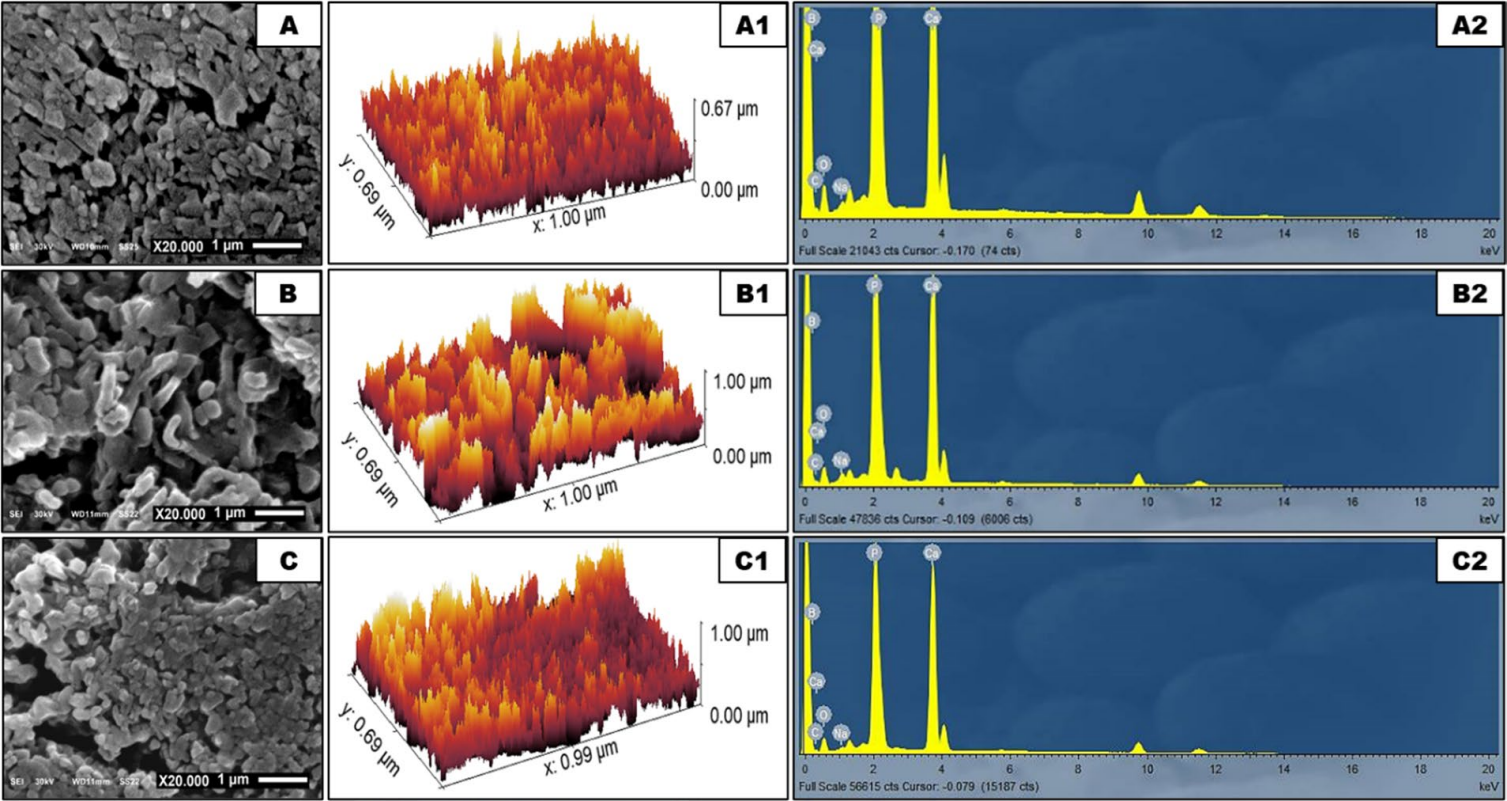
SEM micrographs, their corresponding 3D images, and EDX of MBBGS (**A**, **A1**, **A2**), MBBGS + mHPN (**B**, **B1**, **B2)**, and MBBGS + nHPN (**C**, **C1**, **C2**) after 30 days immersion in SBF.

The prolonged immersion of the three different scaffolds in SBF for 30 days resulted in a dramatic transformation of the surface morphology. The MBBGS surface (Fig. 4A, A1) exhibited the development of an intricate network of interconnected pores and voids, resulting in a highly porous architecture that facilitated interaction between the bioactive glass and SBF. In the EDX analysis (Fig. 4, A2), it exhibited a calcium to phosphate (Ca/P) ratio of ~ 1.04, indicating a composition somewhat different from an ideal stoichiometric HA (1.67).

The surface in MBBGS + mHPN (Fig. 4B, B1) showed the appearance of dense mineral deposit coverage, suggesting possible apatite formation. Some of the rod-like features seemed partially dissolved or integrated into the mineral layer. The porous network still existed, but appeared more interconnected, which might enhance bioactivity. The incorporation of mHPN in the MBBGS in the EDX analysis (Fig. 4B2) resulted in a gradual increase in Ca/P ratio to ~ 1.29, playing a crucial role in enhancing the biomineralization process and optimizing the formation of bone-like apatite layers.

Alternatively, the MBBGS + nHPN surface (Fig. 4C, C1) appeared as densely mineralized, with a more compact and uniform apatite layer. There was less porosity visible, indicating enhanced mineral deposition compared to mHPN. In nHPN, improved nucleation of apatite explained the higher coverage and smoother mineral integration. The evolution of Ca/P ratio (~ 1.45), observed through EDX analysis (Fig. 4C2), revealed significant insights into the bioactive behavior and mineralization process of the MBBGS system incorporating nHPN. This progress towards the ideal Ca/P ratio of stoichiometric HA indicated that HPN was not merely a passive additive but actively contributed to the scaffold’s enhanced bioactive response and its ability to form bone-like mineral phases.

### Evaluation of new bone formation by CBCT (Fig. 5)

**Fig. 5:**
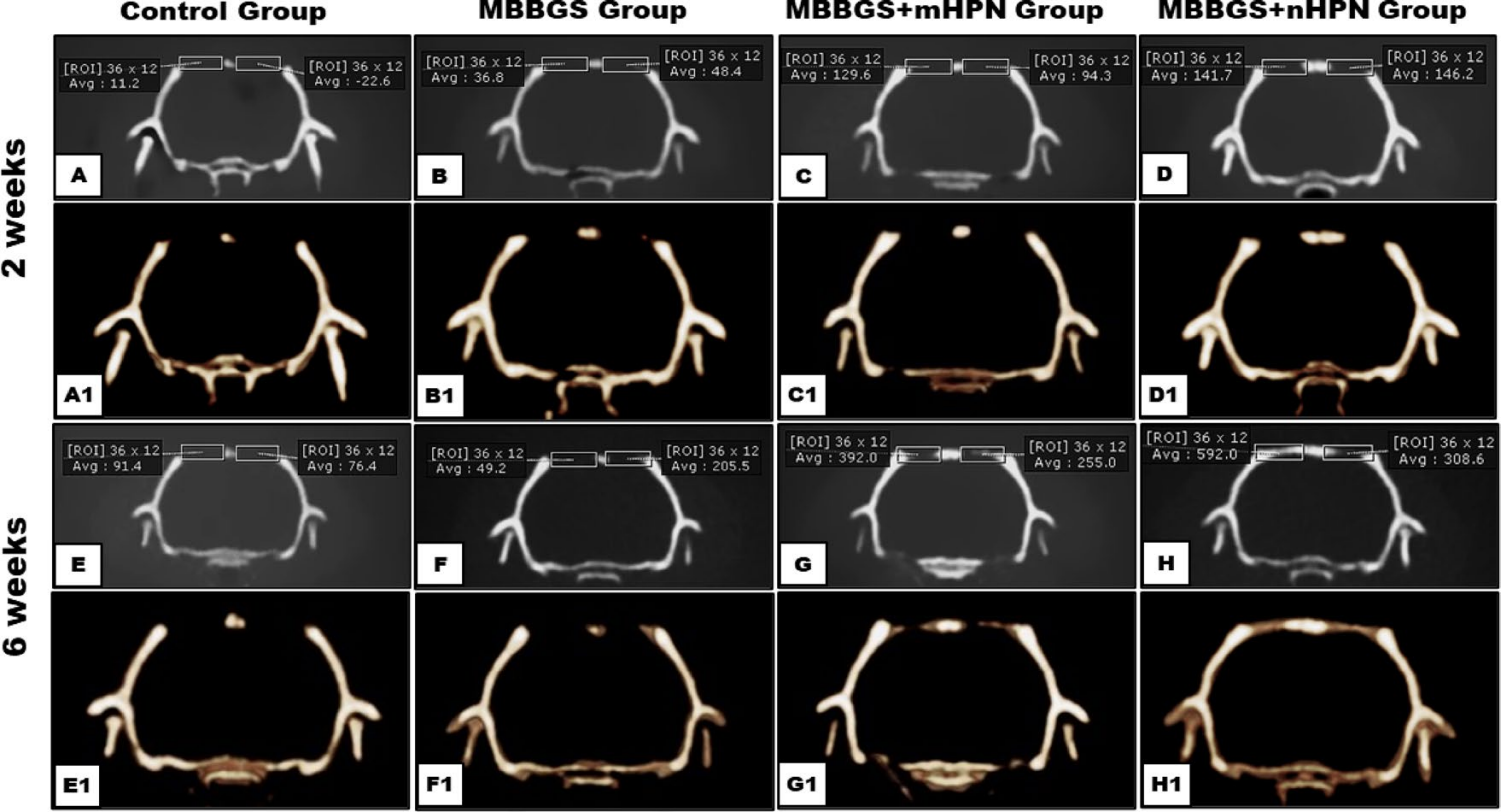
CBCT 2D images and their corresponding 3D images for the control group (**A**,**A1**), MBBGS group (**B**,**B1)**, MBBGS + mHPN group (**C**,**C1**), and MBBGS + nHPN group (**D**,**D1**) at 2 weeks, and for the control group (**E**,**E1**), MBBGS group (**F**,**F1)**, MBBGS + mHPN group (**G**,**G1**), and MBBGS + nHPN group (**H**,**H1**) at 6 weeks.

At 2 weeks postoperatively, CBCT examination showed that the four experimental groups’ early regeneration varied significantly, as indicated by bilateral ROI in Hounsfield Units (HU). The control group (Fig. 5A, A1) showed the least amount of mineralized tissue formation within the calvarial defects, as evidenced by its lowest radiodensity values. On the other hand, the MBBGS group (Fig. 5B, B1) showed a moderate rise in HU values, which was indicative of early mineral deposition and scaffold integration. Radiodensity further increased by adding mHPN to the scaffold (Fig. 5C, C1), indicating better early osteogenic activity. Significantly higher levels of early mineralization were indicated by the highest HU values obtained by the MBBGS + nHPN group (Fig. 5D, D1).

At 6 weeks postoperatively, CBCT revealed a progressive increase in radiodensity across all treatment groups, with the MBBGS + nHPN group (Fig. 5H, H1) exhibiting the highest bone density. This marked enhancement suggested that nHPN exhibited superior osteoinductivity compared to the MBBGS + mHPN group (Fig. 5G, G1), which also demonstrated significant mineralization. The MBBGS group (Fig. 5F, F1) displayed moderate regeneration, while the control group (Fig. 5E, E1) showed the lowest radiodensity, reflecting insufficient natural healing. 

**Fig. 6:**
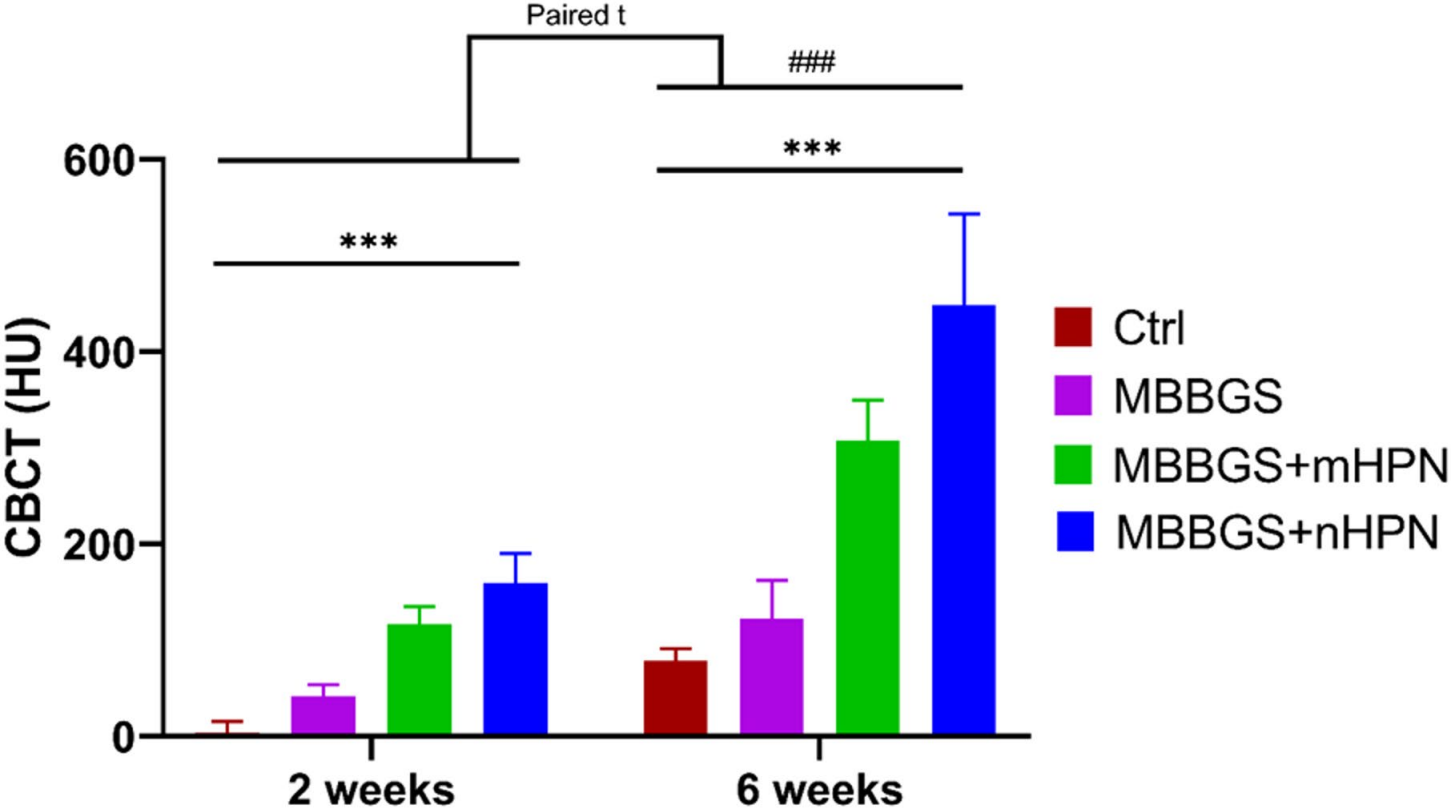
Bar charts represented CBCT values (HU) at 2 and 6 weeks for Control, MBBGS, MBBGS + mHPN, and MBBGS + nHPN groups. Group comparisons at each time point were assessed using two-way ANOVA followed by independent t-tests for intergroup differences (indicated by *), and paired t-tests were used to compare changes within the same group across time points (indicated by #). ***P < 0.0001 and ###*P* < 0.0001.

Statistical analyses of CBCT mean values (Table 1; Fig. 6) showed that all pairwise comparisons between groups were highly significant (*p* < 0.001). Paired analysis confirmed significant increases in bone density over time within each group (all *p* < 0.001). Two-way ANOVA revealed highly significant main effects of group and period, as well as a significant group × period interaction (all *p* < 0.0001), confirming that bone density improved over time in a treatment-dependent manner.

**Table 1:** Means ± SD of CBCT (HU), MTC (%), and OPN (%) at 2 and 6 weeks in the four study groups (control, MBBGS, MBBGS + mHPN, MBBGS + nHPN), with between-group and within-group comparisons.

Variable	Period	*N*	Mean ± SD				*p* value‡							F ratio, *p* value*		
			Control (1)	MBBGS (2)	MBBGS+ mHPN (3)	MBBGS+nHPN (4)	1 × 2	1 × 3	1 × 4	2 × 3	2 × 4	3 × 4	Groups		Periods	Groups×periods
CBCT (HU)	2w	14	3.37 ± 12.11	41.92 ± 11.59	116.87 ± 17.81	159.00 ± 31.39	*p* < 0.0001	*p* < 0.0001	*p* < 0.0001	*p* < 0.0001	*p* < 0.0001	*p* < 0.0001	F = 221.8, *p* < 0.0001		F = 396.1, *p* < 0.0001	F = 38.8, *p* < 0.0001
	6w		78.30 ± 12.62	128.28 ± 44.13	307.41 ± 42.06	447.69 ± 95.09	*p* < 0.001	*p* < 0.0001	*p* < 0.0001	*p* < 0.0001	*p* < 0.0001	*p* < 0.0001				
p value†	2w vs. 6w	–	*p* < 0.0001	*p* < 0.0001	*p* < 0.0001	*p* < 0.0001	–									
MTC (%)	2w	14	0.71 ± 0.14	2.78 ± 0.51	5.68 ± 1.00	9.22 ± 1.42	*p* < 0.0001	*p* < 0.0001	*p* < 0.0001	*p* < 0.0001	*p* < 0.0001	*p* < 0.0001	F = 788.2, *p* < 0.0001		F = 912.2, *p* < 0.0001	F = 74.6, *p* < 0.0001
	6w		2.80 ± 0.22	8.35 ± 0.77	10.56 ± 1.11	19.05 ± 1.59	*p* < 0.0001	*p* < 0.0001	*p* < 0.0001	*p* < 0.0001	*p* < 0.0001	*p* < 0.0001				
p value^†^	2w vs. 6w	–	*p* < 0.0001	*p* < 0.0001	*p* < 0.0001	*p* < 0.0001	–									
OPN (%)	2w	14	0.12 ± 0.07	1.01 ± 0.24	5.33 ± 0.68	8.96 ± 0.87	*p* < 0.0001	*p* < 0.0001	*p* < 0.0001	*p* < 0.0001	*p* < 0.0001	*p* < 0.0001	F = 807.7, *p* < 0.0001		F = 1522.1, *p* < 0.0001	F = 30.7, *p* < 0.0001
	6w		3.81 ± 0.39	8.78 ± 0.73	11.84 ± 1.43	15.92 ± 1.30	*p* < 0.0001	*p* < 0.0001	*p* < 0.0001	*p* < 0.0001	*p* < 0.0001	*p* < 0.0001				
p value†	2w vs. 6w	–	*p* < 0.0001	*p* < 0.0001	*p* < 0.0001	*p* < 0.0001	–									

^†^Paired t-test (2w vs. 6w within group).

^‡^Independent t-test (between groups at the same time point).

*Two-way ANOVA (Group, Period, Group×Period) SD: standard deviation, P: Probability, N: sample number, %: percentage, HU: Hounsfield unit.

### Histological and immunohistochemical results

**Fig. 7:**
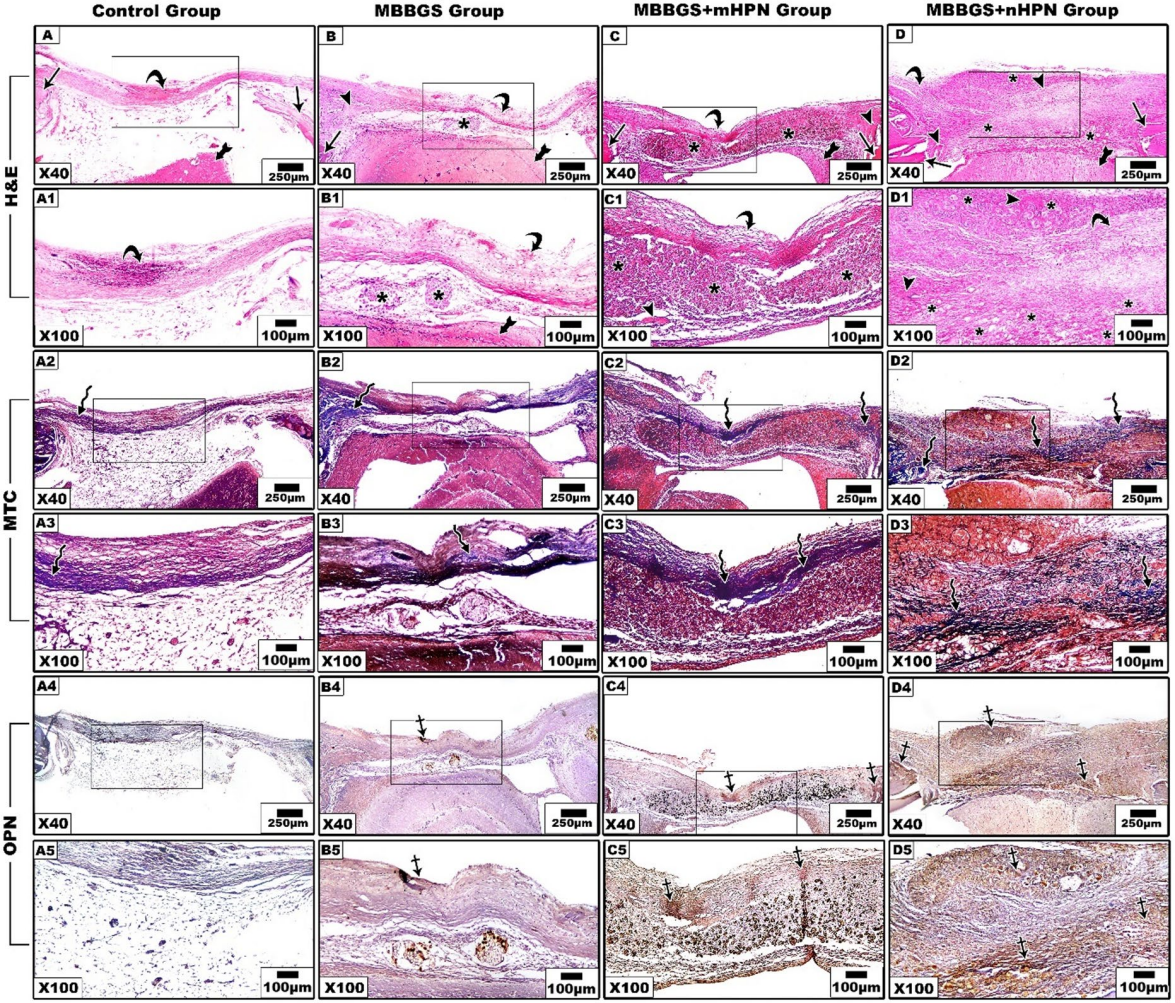
Histological and immunohistochemical stained sections, 2 weeks postoperatively, showed regeneration of rat calvarial bone defects in the four groups. Control group (**A**–**A5**), MBBGS group (**B**–**B5**), MBBGS + mHPN group (**C**–**C5**), MBBGS + nHPN group (**D**–**D5**). H&E-stained sections showed old bone (arrows), new bone (arrow heads), and connective tissue (curved arrow). MTC stain indicated collagen formation (zigzag arrows). The immunohistochemical reaction to OPN (crossed arrows), scaffold (asterisks), and brain (tailed arrow). Representative histological sections were shown at low (×40) and high (×100) magnification. Scale bars as indicated.

Histological findings revealed distinct patterns of tissue response and healing progression among the groups, particularly in the central region of the defects. At 2 weeks postoperatively, H&E staining showed no signs of spontaneous bone regeneration in the control group, with the defect area remaining nearly unfilled by bone and containing only loosely arranged fibrous connective tissue (Fig. 7A,A1). In MTC-stained sections, a small amount of loose granulation tissue was observed within the defect area that had blue-stained collagen fibers (Fig. 7A2, A3). It displayed almost a negative reaction to immunostaining in OPN-stained sections (Fig. 7A4,A5).

The MBBGS group showed persistent fibrous tissue infiltration with minimal osteogenic activity, suggesting that bioglass alone initiated a limited early healing response (Fig. 7B and BB1). Additionally, it displayed a slight increase in collagen fibers, but these were still somewhat sparse and haphazard, indicating very little healing activity (Fig. 7B2,B3). Notably, it revealed minimal reaction to OPN (Fig. 7B4,B5).

In the MBBGS + mHPN group, there were modest signs of osteoid formation, indicating some stimulatory effect from mHPN, as well as the presence of the scaffold deposits implying active drug release, likely contributing to osteoinductivity and anti-inflammatory effects (Fig. 7C,C1). Also, it demonstrated denser collagen fiber bundles, indicating an accelerated matrix maturation (Fig. 7C2,C3). Besides, the sections showed elevated OPN positivity (Fig. 7C4,C5).

In contrast, when the bioglass was loaded with nHPN, it demonstrated enhanced cellular activity, denser granulation tissue, and early signs of new bone islands (Fig. 7D, D1). It also exhibited the highest collagen content, with thicker and more intensely stained fibers (Fig. 7D2,D3). Notably, it displayed more intense OPN immunopositivity (Fig. 7D4, D5).

**Fig. 8:**
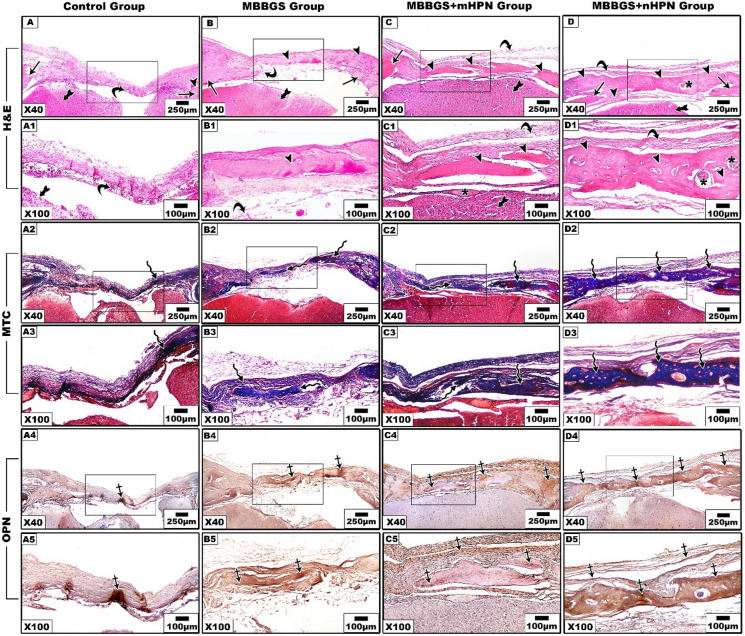
Histological and immunohistochemical stained sections, 6 weeks postoperatively, showed regeneration of rat calvarial bone defects in the four groups. Control group (**A**–**A5**), MBBGS group (**B**–**B5**), MBBGS + mHPN group (**C**–**C5**), MBBGS + nHPN group (**D**–**D5**). H&E-stained sections showed old bone (arrows), new bone (arrow heads), and connective tissue (curved arrow). MTC stain indicated collagen formation (zigzag arrows). The immunostaining reaction to OPN (crossed arrows). Scaffold (asterisks). Brain (tailed arrow). Representative histological sections were shown at low (×40) and high (×100) magnification. Scale bars as indicated.

At 6 weeks, clear distinctions between scaffold degradation and regeneration of bone were observed. The control group was still unable to show substantial regeneration or bone bridging, with only a small amount of new osteoid development emerging along the borders of the dense connective tissue (Fig. 8A, A1). Also, it displayed a slight increase in collagen fibers, but these remained relatively sparse and disorganized, reflecting delayed healing (Fig. 8A2, A3). In OPN-stained sections, they exhibited minimal expression to immunostaining (Fig. 8A4, A5).

The MBBGS group showed irregular trabeculae of newly formed woven bone extending inward from the defect margins, and the scaffold was almost completely degraded. The central region of the defect displayed some bridging bone with moderate cellularity (Fig. 8B and B1). Additionally, it showed a significant rise in collagen deposition with better-organized bundles, which is indicative of partial bone matrix development (Fig. 8B2, B3). Notably, it displayed a greater increase in OPN expression (Fig. 8B4, B5).

The MBBGS + mHPN group exhibited more mature bone trabeculae and better organization, with reduced scaffold residues (Fig. 8C, C1). Also, in MTC-stained sections, extensive collagen deposition and organized structures were clear (Fig. 8C2, C3). Furthermore, the reaction was mostly expressed to OPN as shown in Fig. 8C4, C5.

The most significant regeneration was seen in the MBBGS + nHPN group, where mature bone structures with embedded osteocytes, prominent marrow spaces, and well-integrated vascular networks were present. Interestingly, more scaffold residues were still observed, as compared to other groups, indicating persistent drug bioavailability after 6 weeks and sustained release, maintaining the regenerative process (Fig. 8D and DD1). In MTC-stained sections, they displayed the most pronounced collagen maturation, densely thick and aligned fibers (Fig. 8D2, D3). Evidently, it revealed the most robust immunostaining reaction to OPN (Fig. 8D4, D5).

### Histomorphometric analysis

**Fig. 9:**
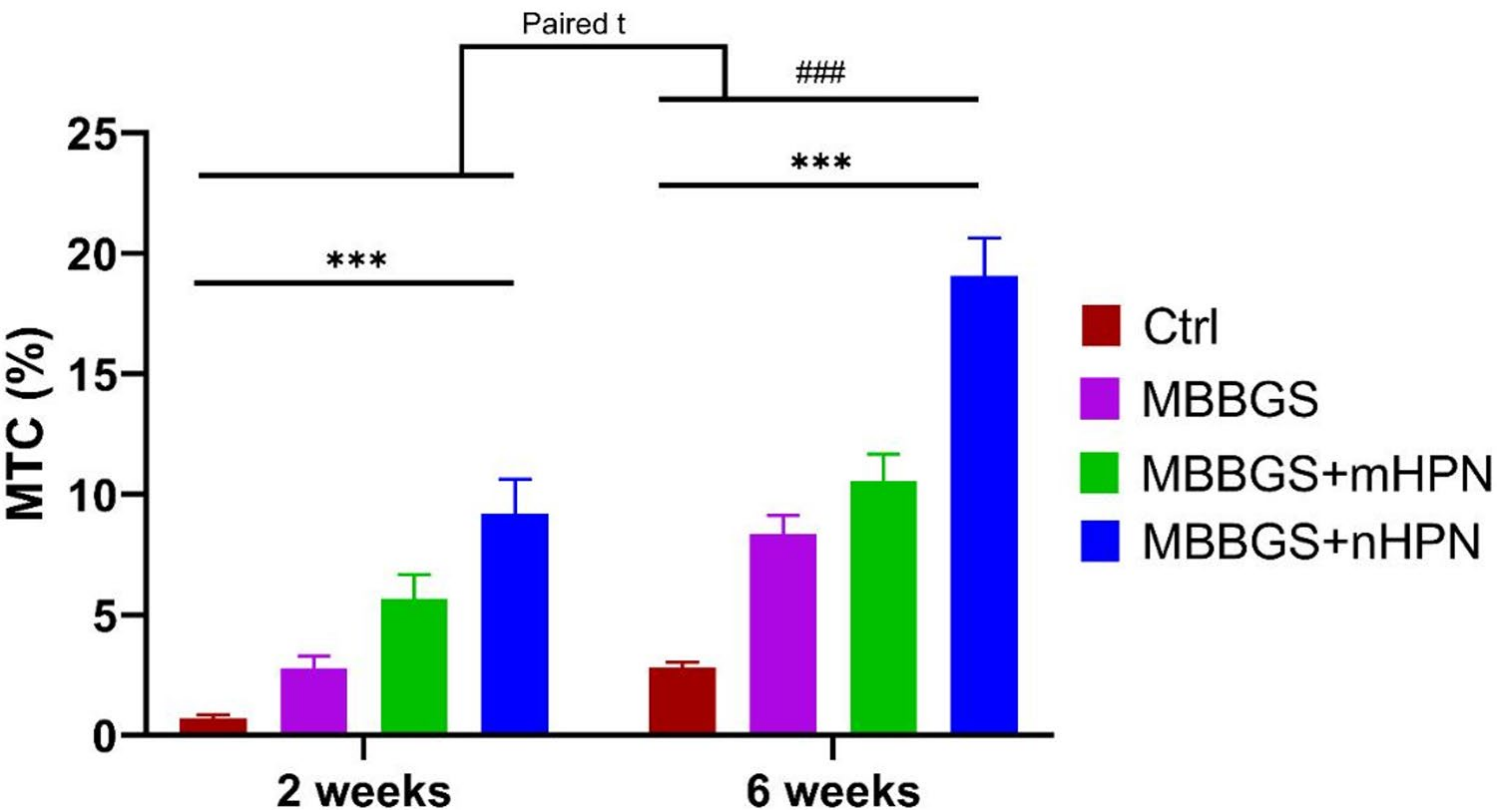
Bar charts represented mean values of MTC area percentage at 2 and 6 weeks in Control, MBBGS, MBBGS + mHPN, and MBBGS + nHPN groups. Group comparisons at each time point were analyzed by two-way ANOVA followed by independent t-tests for intergroup differences (indicated by *), and paired t-tests were used to compare each group between 2 and 6 weeks (indicated by #). ***P < 0.0001 and ###*P* < 0.0001.

At 2 weeks, mean values of collagen fibers stained by MTC followed the order: Control < MBBGS < MBBGS + mHPN < MBBGS + nHPN, with all between-group comparisons statistically significant. Over the 6 weeks, this trend was maintained, with all groups showing further improvement, and MBBGS + nHPN showing the highest collagen levels. Time comparisons within groups demonstrated highly significant differences in 6 weeks compared with 2 weeks, confirmed by paired testing (all *p* < 0.001). Two-way ANOVA showed significant effects of group, period, and their interaction (all *p* < 0.0001), supporting a strong enhancement of calcification with both time and treatment (Table 1; Fig. 9).

**Fig. 10:**
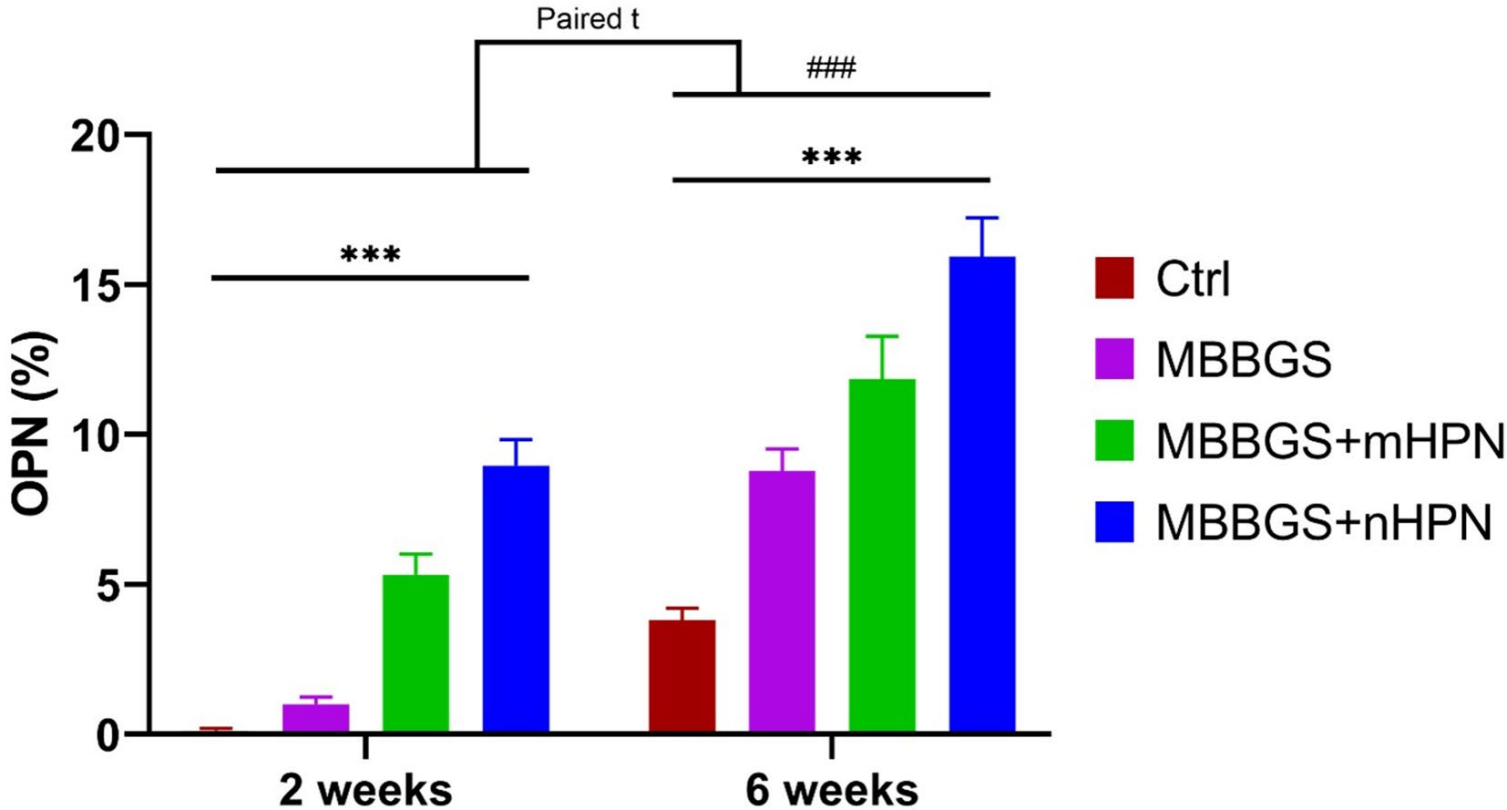
Bar charts represented mean values of OPN area percentage at 2 and 6 weeks in Control, MBBGS, MBBGS + mHPN, and MBBGS + nHPN groups. Group comparisons at each time point were analyzed using two-way ANOVA followed by independent t-tests for intergroup differences (indicated by *), and paired t-tests were performed to assess changes within each group between 2 and 6 weeks (indicated by #). ***P < 0.0001 and ###*P* < 0.0001.

When transitioning to OPN expression for 2 weeks, mean values were lowest in the control group and progressively higher in MBBGS, MBBGS + mHPN, and MBBGS + nHPN groups. At 6 weeks, OPN levels increased further in all groups, with the MBBGS + nHPN group showing the highest expression. Paired analysis showed significant increases in OPN expression over time within each group (all *p* < 0.001). Two-way ANOVA confirmed strong main effects of group and period and a significant group × period interaction (all *p* < 0.0001), supporting that OPN expression was strongly dependent on both time and treatment, with the greatest effect observed in the nHPN-enhanced group (Fig. 10).

## Discussion

Nanomedicine has been introduced in regenerative medicine as a promising approach for elevating the characteristics of therapeutic substances. In most situations, the fabrication of nanoscale particles of different ingredients can improve their properties^41^. In the present study, the MBBGS + nHPN group demonstrated enhanced Ca/P ratio and more uniform apatite formation compared with MBBGS + mHPN and MBBGS groups. In vivo, CBCT and histological analyses revealed increased mineral deposition and well-integrated scaffold-tissue interfaces in the MBBGS + nHPN group, consistent with the in vitro mineralization trend. Overall, these results highlighted a synergistic effect of nanoscale scaffold architecture and bioactive flavonoid on promoting early osteogenesis.

CSD is one of the most widely used experimental models for evaluating the regeneration potential of biomaterials due to its physiological resemblance to human bone remodeling^42^.

While preclinical model selection typically considered the phylogenetic tree, the use of small animals, like rodents, was acceptable when reliable and scientifically valid data were obtained^43^. This supported translational relevance for craniofacial bone repair applications. The calvarial bone was selected in this study to overcome the small size and anatomical complexity of the alveolar bone, as it shares developmental characteristics with the maxilla and mandibular body^44^. A 5 mm defect was considered a CSD in young adult rats, as confirmed by Hudieb et al.^45^.

Sol-gel glasses have a mesoporous texture with nanometer-sized pores, and a high surface area compared with melt-derived glasses^46^. The architecture of scaffolds used in the present study was fabricated into a “trabecular” with a porosity that supported tissue ingrowth, cell attachment, and diffusion of nutrients into the defect site, providing characteristics favorable for bone ingrowth^47^. Importantly, the surface of scaffolds was further modified at the nanoscale, enhancing surface roughness and bioactivity, compared with conventional types^48,49^. This nanoscale modification represented an advancement over previously reported bioglass scaffolds.

The results of a previous study by Sepulveda et al. showed that the dissolution behavior of sol-gel glasses depends more on the mesoporous texture than on the particle size^50^. The degradation of the scaffolds in our study followed the trend MBBGS + nHPN < MBBGS + mHPN < MBBGS, indicating that the incorporation of HPN, particularly in the nanoscale form, contributed to a more controlled dissolution. This slower degradation likely facilitated sustained local release of HPN, providing a foundation for enhanced mineralization and scaffold-tissue integration.

HPN presents a compelling candidate for enhancing bone regeneration by stimulating osteoblast differentiation^51^. However, its therapeutic application is limited due to low oral bioavailability (< 25%), low solubility in aqueous solutions, and physicochemical instability. Thus, topical delivery enables site-directed administration, reducing total drug use and maintaining the drug concentration at the site of action^52^. In this study, HPN was added after sintering via the impregnation method to avoid the high processing temperatures that could degrade the drug, offering better control over drug loading efficiency and stability^53^.

CBCT is frequently used in dental practice and has proven to be a reliable non-invasive modality for bone regeneration since it offers sufficient resolution with less radiation and shorter acquisition time than CT^54,55^. Our study used CBCT to assess progressive relative mineralization reflecting bone formation during healing, relying on a semi-quantitative tracking of grayscale changes with consistent imaging settings and ROI placement^56^. The visibility of scaffolds in CBCT imaging depends on many factors, such as porosity, intrinsic low radiopacity, particularly in forms produced from sol-gel, thin scaffold (1 mm), post-implantation coatings of biological materials, and progressive bone healing, which obscure their detection^57^.

Histological evaluation postoperatively revealed distinct differences in the healing response among the four groups, as in the control group, indicating minimal or no mineralized tissue formation within the calvarial defects. In contrast, the group treated with MBBGS alone demonstrated more scaffold integration and mineral deposition. This result was supported by the findings of Zhang et al., who found that bioactive glass scaffolds containing boron exhibited good biocompatibility as their pore shape enhanced cell proliferation and offered transport pathways for nutrients and metabolites needed for cell growth, leading to bone regeneration^58^.

Notably, when HPN was loaded on the bioglass group, it demonstrated improved scaffold stability and early fibrous tissue ingrowth, which agreed with Eliwa et al., who described that HPN accelerated hard tissue formation^59^. These results were consistent with earlier research since Liu et al. showed that early phase osteoinduction is enhanced by surface alterations or drug inclusion into borate scaffolds^60^. Also, Aboshosha et al. found that HPN exerted a protective effect on alveolar bone osteoporosis in diabetic rats, and this echoed our study^61^.

The MBBGS + nHPN group demonstrated the most advanced healing features, including matrix deposition and well-integrated scaffold-tissue interfaces. This was attributed to the particle-size-dependent effects of HPN, where small particles provided higher surface area and better distribution, enhancing cell adhesion, proliferation, and differentiation compared to larger HPN particles^62^. Additionally, this aligned with Ali et al., who showed that the modified nHPN exhibited cytocompatibility and increased potency compared to native HPN^63^. Another study by Alherz et al. revealed that HPN nanoparticle treatment diminished renal toxicity by alleviating inflammation and reducing apoptosis^64^. The scarcity of literature on HPN particle-size effects on bioglass-based bone regeneration emphasized the significance of our comparative study.

When transitioning to OPN, HPN-loaded MBBGS showed a stronger reaction to immunohistochemical staining than MBBGS and the control groups. This was consistent with a previous study by Miguez et al., who showed that HPN could improve osteogenesis by increasing osteogenic markers and enhancing the composition and structure of the bone matrix^65^. Besides, Hong et al. had shown that HPN might raise the alkaline phosphatase content of osteoblasts and effectively suppress osteoclast development, and expressions of osteoclast-specific markers in a dose-dependent manner^66^.

The improved osteogenic response associated with nHPN could be explained by multiple complementary mechanisms. Nanoscale formulation enhanced cellular uptake of HPN, enabling more efficient internalization by osteoblasts and mesenchymal stem cells and thereby potentiating osteogenic signaling pathways^67^. In addition, loading of nHPN on MBBGS allowed sustained and controlled release of bioactive compounds, providing prolonged osteoinductivity stimulation^68^. Importantly, the intrinsic antioxidant properties of HPN contributed to osteoinduction by reducing oxidative stress, which is known to impair osteoblast differentiation and matrix mineralization^69^.

Despite the promising results, this study has some limitations, as the follow-up period was relatively short, and the CBCT analysis relied on a semi-quantitative approach. Future studies with extended observation periods and alternative imaging modalities are necessary to validate and expand upon these findings.

## Conclusion

According to the previous results of this study, we could highlight the importance of both scaffold composition and drug formulation in promoting bone regeneration. Borate-based bioglass provided a degradable framework that facilitated tissue ingrowth, while the addition of HPN significantly enhanced osteogenic activity. The nanoform of the drug proved that it was more effective than the microform, likely due to its increased surface area, cellular uptake, and bioavailability. Collectively, the combination of nHPN and MBBGS demonstrated the most promising regenerative outcomes in this CSD model.

## Supplementary Information

Below is the link to the electronic supplementary material.

Supplementary Material 1

## Data Availability

All data generated or analyzed during this study are included in this published article.

## Abbreviations

ANOVA: Analysis of variance
CSD: Critical-sized defect
CBCT: Cone beam computed tomography
2D: Two dimensions
3D: Three dimensions
EDTA: Ethylenediaminetetraacetic acid
EDX: Energy dispersive X-ray
Fig: Figure
FTIR: Fourier transform infrared
H&E: Hematoxylin and Eosin
HA: Hydroxyapatite
HPN: Hesperidin
HU: Hounsfield unit
mHPN: Micro-sized form of hesperidin
MBBGS: Modified borate bioglass scaffold
MERC: Mansoura Experimental Research Centre
MTC: Masson’s trichrome
nHPN: Nano-sized form of hesperidin
OPN: Osteopontin
PBS: Phosphate-buffered solution
ROI: Region of interest
SD: Standard deviation
SBF: Simulated body fluid
SEM: Scanning electron microscope
XRD: X-ray diffraction
