# Supplementary material for: Impact of two different hesperidin forms loaded on nanoscale modified borate bioglass scaffolds on rat critical-sized calvarial defects
Source: Sci Rep. 2026 Feb 3;16:4777. doi: 10.1038/s41598-026-35881-z (PMC12873398; doi:10.1038/s41598-026-35881-z)
Supplement: Supplementary file 1 — Supplementary Material 1 [file 41598_2026_35881_MOESM1_ESM.pdf]

Table S1: Chemicals for *in vitro* experiments

Materials	Supplier
Boric acid (H_3BO_3)	EDWIC Co.
Phosphoric acid (H_3PO_4)	Sigma-Aldrich
Calcium nitrate tetrahydrate ($Ca(NO_3)_2 \cdot 4H_2O$)	EDWIC Co.
Sodium nitrate ($NaNO_3$)	El-Nasr Chemicals Co.
Hesperidin (HPN)	Alfa Aesar, Thermo Fisher Scientific Co.
A simulated body fluid (SBF)	Alfa Chemical Group

Table S2: Equipment and manufactures

Item	Manufacturer addresses
Scanning Electron Microscope (SEM)	JSM-6510LV, JEOL, Tokyo, Japan
X-ray Diffractometer (XRD)	PAN analytical X' Pert PRO, Almelo, The Netherland
Fourier Transform Infrared (FTIR) spectroscope	Nicolet iS10 Model, Thermo Fisher Scientific, USA
Energy Dispersive X-ray (EDX) spectrometer	Oxford Xmax^{50}, Abingdon, UK
Cone Beam Computed Tomography (CBCT) machine	I-CAT ISI, Hatfield, USA
Trephine Bur (5 mm diameter)	Medesy®, Italy
Low-speed Contra-Angle Dental Handpieces	Sirona®, Germany
Digital Camera	ToupCam®, model no.: XCAM1080PHA, ToupTek Photonics, Hangzhou, China
Light Microscope	Olympus® CX22, Tokyo, Japan

Specimen Preparation for SEM Analysis

- Specimens were immersed in the standard Karnovsky's fixative (2.5% glutaraldehyde, 2% formaldehyde, 0.02% sodium azide in 0.05 M Na-cacodylate buffer at pH 7.4).
- Dehydration was performed using graded ethanol series thoroughly rinsed with phosphate-buffered solution (PBS), followed by air drying.
- Finally, specimens were sputter-coated with a thin layer of gold prior to SEM examination.

Table S3: Medications

Drug	Dose	Route
ketamine hydrochloride	80 mg/kg	IP
Xylazine hydrochloride	10 mg/kg	IP
Amoxicillin	200 mg/kg twice daily	IM
ketoprofen	5 mg/kg once daily	SC
Sodium thiopental	120 mg/kg	IP

Table S4: Stains and antibodies

Reagent / Kit	Supplier	Catalog No
Hematoxylin and Eosin (H&E) high-definition constant staining kit	Servicebio®, China	G1076
Masson's Trichrome (MTC) staining Kit	Servicebio®, China	G1006
Anti-OPN primary antibody	Servicebio®, China	GB11500
Anti-Mouse/Rabbit PolyVue^{TM} Plus HRP/DAB Detection System kit	Diagnostic Biosystems, USA	-

Immunohistochemical Staining Procedure for Osteopontin (OPN)

- Sections were incubated with a primary anti-OPN antibody at the recommended dilution: 1:500.
- Slides were incubated with an avidin-enzyme conjugated for 10 minutes and washed with PBS.
- Diaminobenzidine (DAB) was applied as the chromogen for 5 minutes at room temperature.
- Slides were counterstained with Mayer's haematoxylin.
- Sections were dehydrated and mounted on positively charged slides for microscopic examination.
